# Exfoliation of Al-Residual Multilayer MXene Using Tetramethylammonium Bases for Conductive Film Applications

**DOI:** 10.3389/fchem.2022.841313

**Published:** 2022-03-21

**Authors:** Emi Saita, Masaki Iwata, Yuki Shibata, Yuki Matsunaga, Rie Suizu, Kunio Awaga, Jun Hirotani, Haruka Omachi

**Affiliations:** ^1^ Department of Electronics, Graduate School of Engineering, Nagoya University, Nagoya, Japan; ^2^ Department of Chemistry, Graduate School of Science, Nagoya University, Nagoya, Japan; ^3^ PRESTO, Japan Science and Technology Agency, Saitama, Japan; ^4^ Research Center for Materials Science, Nagoya University, Nagoya, Japan

**Keywords:** MXene, exfoliation, tetramethylammonium base, Al etching, transparent conductive film

## Abstract

This study describes the concise exfoliation of multilayer Ti_3_C_2_T_*x*_ MXene containing residual aluminum atoms. Treatment with tetramethylammonium base in a co-solvent of tetrahydrofuran and H_2_O produced single-layer Ti_3_C_2_T_*x*_, which was confirmed *via* atomic force microscopy observations, with an electrical conductivity 100+ times that of Ti_3_C_2_T_*x*_ prepared under previously reported conditions. The scanning electron microscopy and X-ray diffraction measurements showed that the exfoliated single-layer Ti_3_C_2_T_*x*_ MXenes were reconstructed to assembled large-domain layered films, enabling excellent macroscale electric conductivity. X-ray photoelectron spectroscopy confirmed the complete removal of residual Al atoms and the replacement of surface fluorine atoms with hydroxy groups. Using the exfoliated dispersion, a flexible transparent conductive film was formed and demonstrated in an electrical application.

## Introduction

Since first reported by Naguib et al. (2011), MXenes have attracted wide attention as novel analogs of two-dimensional layer nanomaterials such as graphene, hexagonal boron nitride, and transition-metal dichalcogenides. MXenes show high mechanical durability and transparency conferred by the layer structure, along with unique chemical natures and excellent electrical characteristics (Naguib et al., 2014; Verger et al., 2019; Wang et al., 2020). The general formula of MXenes is M_*n*+1_X_*n*_T_*x*_ (M: early transition metals, X: carbon and/or nitrogen, T: a terminal functional group such as F, OH, O). The electronic band structures of MXenes depends on the combination of their M, X, and T atoms. For instance, the simple titanium carbide family (e.g., Ti_2_CT_*x*_, Ti_3_C_2_T_*x*_) shows a metallic property with high charge-carrier density, whereas Cr_2_TiC_2_F_2_ and Cr_2_TiC_2_(OH)_2_ are thought to behave as semiconductors. Moreover, MXenes with hydrophilic terminal functional groups are dispersible in water and polar organic solvents without surfactants; accordingly unlike other nanomaterials, they can act as scaffolds for chemical modification. These advantages are expected to be exploited in a wide range of applications, such as Li- or Na-ion batteries (Naguib et al., 2012; Liang et al., 2015; Natu et al., 2018), transparent conductive films (Dillon et al., 2016; Hantanasirisakul et al., 2016; Zhou et al., 2021), catalysts for hydrogen evolution (Seh et al., 2016; Intikhab et al., 2019) and supercapacitors (Lukatskaya et al., 2013; Dall’Agnese et al., 2015; Wang et al., 2015).

Typically, MXenes are produced from the MAX phase (M_*n*+1_AlX_*n*_) in a hydrogen fluoride (HF) treatment, which removes the atomically thin layer of aluminum atoms (Naguib et al., 2011; Lukatskaya et al., 2013; Luo et al., 2016). To date, researchers can easily access commercially available MXenes, without using a hazardous HF acid (the highest hazard level in the health section of NFPA 704). However, these MXenes maintain their multilayer structure *via* covalent bonding between the layers and residual Al atoms. Such MXenes are rarely dispersed and immediately precipitate out even after vigorous sonication. Furthermore, films prepared from filtrates of these MXenes are electrically non-conductive. To realize the above applications, conductive liquid-state MXene materials are desired for fabrication processes such as spray coating, spin cast, and filtration. Therefore, a concise and HF-free method that exfoliates and disperses MXene as a single-layer nanomaterial by removing the Al atoms is required.

This work reports on the exfoliation of Al-residual multilayer Ti_3_C_2_T_*x*_, the most commonly used conductive MXene, with organic bases containing a tetramethylammonium cation. This treatment effectively removes the interlayer Al atoms from the MXenes. The Ti_3_C_2_T_*x*_ exfoliated in a co-solvent of tetrahydrofuran (THF) and H_2_O exhibited superior electrical conductivity. We also demonstrate the application of flexible transparent conductive films fabricated by spray-gun coating with the exfoliated Ti_3_C_2_T_*x*_ dispersion.

## Experimental Section

### Sample Preparation

Ti_3_C_2_T_*x*_ MXene (Japan Material Technologies Corporation, 4 mg), 0.2 mmol of tetramethylammonium reagent (Table 1), and 2 ml of solvent were added to a vial containing a magnetic stirring bar. After stirring at room temperature for 24 h, the mixture was transferred to a 15-ml conical tube with deionized (DI) water (0.2 ml × 4), which were degassed by N_2_ bubbling for 30 min prior to use. After adding 2-propanol (2 ml), the mixture was centrifuged at 4,500 rpm for 5 min and the supernatant was removed. After adding 2 ml of DI water to the sediment, the mixture was dispersed in a bath-sonicator (BM EQUIPMENT, Nanoruptor NR-350) for 40 min and centrifuged at 433 *g* for 30 min. The resulting supernatant was collected as the MXene dispersion. The exfoliation yield was estimated from the intensity of the absorption spectrum at 800 nm, recorded on an ultraviolet–visible spectrophotometer (JASCO V-770) after freeze-drying treatment with EYELA FDS-1000.

**TABLE 1:** Optimized reaction conditions for exfoliating multilayer Ti_3_C_2_T_*x*_ MXene.

Entry	Reagent	Solvent	Time [h]	Yield [%]	*R*s (SD) [Ω/sq.]
1	Me_4_NF·4H_2_O	DMSO	24	22	1.4 × 10^4^ (2.1 × 10^3^)
2	Me_4_NOAc	DMSO	24	4	3.8 × 10^4^ (5.9 × 10^3^)
3	Me_4_NOH·5H_2_O	DMSO	24	12	7.1 × 10^3^ (9.1 × 10^2^)
4	Me_4_NCl	DMSO	24	<1	—
5	Me_4_NBr	DMSO	24	<1	—
6	Me_4_NBF_4_	DMSO	24	<1	—
7	Me_4_NOH·5H_2_O	H_2_O	24	43	2.0 × 10^4^ (9.1 × 10^2^)
8	Me_4_NOH·5H_2_O	THF	24	15	2.0 × 10^3^ (2.5 × 10^2^)
9	Me_4_NOH·5H_2_O	CH_3_CN	24	<1	—
10	Me_4_NOH·5H_2_O	NMP	24	<1	—
11	Me_4_NOH·5H_2_O	CH_3_OH	24	<1	—
12	Me_4_NOH·5H_2_O	THF/H_2_O^a^	24	46	2.0 × 10^3^ (2.5 × 10^2^)
13	Me_4_NOH·5H_2_O	THF/H_2_O^a^	72	59	1.4 × 10^3^ (93)
14^b^	Me_4_NOH·5H_2_O	THF/H_2_O^a^	72	5	2.7 × 10^2^ (6.2)
15^b^	Me_4_NOH·5H_2_O	THF/H_2_O^a^	120	20	1.5 × 10^2^ (8.8)

a Solvent ratio (v/v): THF/H_2_O = 10/1.

b Without sonication treatment.

The sheet resistances (*R*_s_, Ω/sq) of the films were measured using the four-probe method with a Loresta-AX resistivity meter (MCP-TP06P, Mitsubishi Chemical Analytech). Thick MXene films were prepared by vacuum filtration of the dispersions (0.1 mg in 50 ml DI water) on a MF-Millipore 47-mm MCE membrane filter (0.05-µm pore size).

### Characterization

The optical absorption spectra of the dispersion sample were recorded on an ultraviolet–visible spectrophotometer (V-770, JASCO). Raman spectra in the radial-breathing mode region were recorded on an inVia Raman Microscope (Renishaw) excited by 785-nm laser light. A single-monochromator micro-Raman spectrometer was employed in the back-scattering configuration. The sample dispersions were drop-casted onto a silicon wafer before the measurement. X-ray photoelectron spectroscopy (XPS) data were obtained using an ESCALAB XI + spectrometer (Thermo Fisher Scientific) using 300 W Al–Ka radiation. To exclude the substrate signals, a highly concentrated MXene dispersion was drop-coated several times onto a Si/SiO_2_ wafer to form a thick MXene film (>10 nm). Atomic force microscopy (AFM) measurements were acquired using a Dimension Fastscan AFM with a NanoScope V stage controller (Bruker). The samples for AFM observation were prepared by spin-coating the Si/SiO_2_ wafer with 10 µl of the MXene dispersion at 400 rpm for 60 s, followed by 1,000 rpm for 60 s and 1,600 rpm for 60 s. Scanning electron microscopy (SEM) measurements were conducted in an S-4300 (Hitachi) or an ETHOS NX5000 (Hitachi). X-ray diffraction (XRD) spectra were obtained by a SmartLab (Rigaku) through Cu Kα radiation.

### Fabrication of Flexible Transparent Conductive Films

Polyethylenenaphthalete (PEN) films with a thickness of 100 μm and DuPont Films (Q65FA) were surface-treated with a plasma cleaner (YAMATO PR500) at 100 W for 10 min. The MXene dispersion diluted with methanol was coated using a spray-gun (ANEST IWATA HP-TR1). The spray-coated films were dried at 30°C for 16 h under vacuum conditions (<5 Pa). The transparency was determined by the absorption spectra recorded on an ultraviolet–visible spectrophotometer (V-770, JASCO). The sheet resistance (*R*_s_, Ω/sq) of the films was measured using the four-probe method with a Loresta-AX resistivity meter (MCP-TP06P, Mitsubishi Chemical Analytech).

## Results and Discussion

### Optimization of Exfoliation Conditions

**FIGURE 1:**
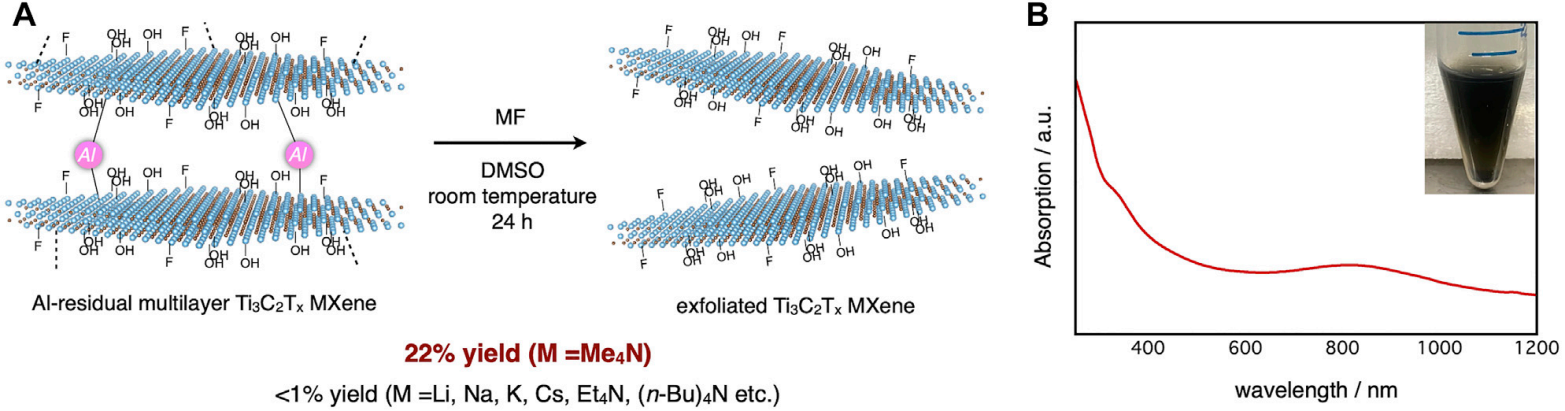
**(A)** Exfoliation of Al-residual Ti_3_C_2_T_*x*_ MXene with Me_4_NF; **(B)** Optical absorption spectrum (main) and typical photograph (inset) of Ti_3_C_2_T_*x*_ MXene dispersion.

Initially, etching reagents other than HF acid were explored for removing residual Al atoms from Ti_3_C_2_T_*x*_ MXene. Prior to the investigation, we tested dimethyl sulfoxide (DMSO) (Mashtalir et al., 2013) and tetrabutylammonium cations (Naguib et al., 2015) as intercalation agents that can expand the inter layer distance of MXenes. These agents did not exfoliate the Al-residual multilayered Ti_3_C_2_T_*x*_ MXene. After screening a variety of organic and inorganic fluoride reagents, it was found that only tetramethylammonium fluoride (Me_4_NF) in DMSO solvent obtained the desired MXene dispersion in 22% yield (Figure 1A). The optical absorption spectrum of the dispersion exhibited a peak around 800 nm, consistent with reported examples (Li et al., 2017) (Figure 1B). Because tetramethylammonium hydroxide (Me_4_NOH) can etch the MAX phase (Xuan et al., 2016; Yang et al., 2018), we supposed that the tetramethylammonium cations were suitably sized to penetrate the inter layers of MXenes, which were geometrically restricted by bonding with aluminum atoms.

Next, we investigated the effect of the counter anions on tetramethylammonium salts (Table 1). Adding Me_4_NOH and tetramethylammonium acetate (Me_4_NOAc) to Me_4_NF enabled the exfoliation of Ti_3_C_2_T_*x*_ MXene, implying that basic counter anions are required for removing the aluminum atoms (entries 1–6). The sheet resistance of the film fabricated with the Me_4_NOH dispersion was 7.1 × 10^3^ Ω/sq., lower than those of the films produced from Me_4_NF-exfoliated and Me_4_NOAc-exfoliated MXenes (1.4 × 10^4^ and 3.8 × 10^4^ Ω/sq., respectively). At this stage, we selected Me_4_NOH as the etching reagent and screened several solvents to improve the exfoliation efficiency and electrical conductivity. When the reaction was performed in aqueous solution under conditions similar to the reported etching conditions of MAX phases, the exfoliation reached 43% yield, but the film conductivity reduced to an undesirable 2.0 × 10^4^ Ω/sq. (entry 7 of Table 1). The exfoliation yield was only 15% in tetrahydrofuran (THF) solvent and was less than 1% in the other organic solvents (entries 8–11 in Table 1). The sheet resistance of the exfoliated MXene (2.0 × 10^3^ Ω/sq.) was three times lower in THF than in DMSO. Surprisingly, a mixed solvent of THF and H_2_O was effective for both exfoliation (46% yield) and conductivity (2.0 × 10^3^ Ω/sq.). Extending the reaction time also enhanced the exfoliation yield (entries 12 and 13 in Table 1). Under these conditions, MXenes were spontaneously exfoliated without the sonication treatment, and the sheer resistance was drastically decreased to ca. 2.0 × 10^2^ Ω/sq. (entries 14 and 15 in Table 1). Eventually, we found that reacting MXene with Me_4_NOH in THF/H_2_O solvent for 120 h produced the desired MXene dispersion, balancing the exfoliation efficiency with high conductivity (entry 15 in Table 1).

### Microscopic Observations and Assembled Structural Analysis

**FIGURE 2:**
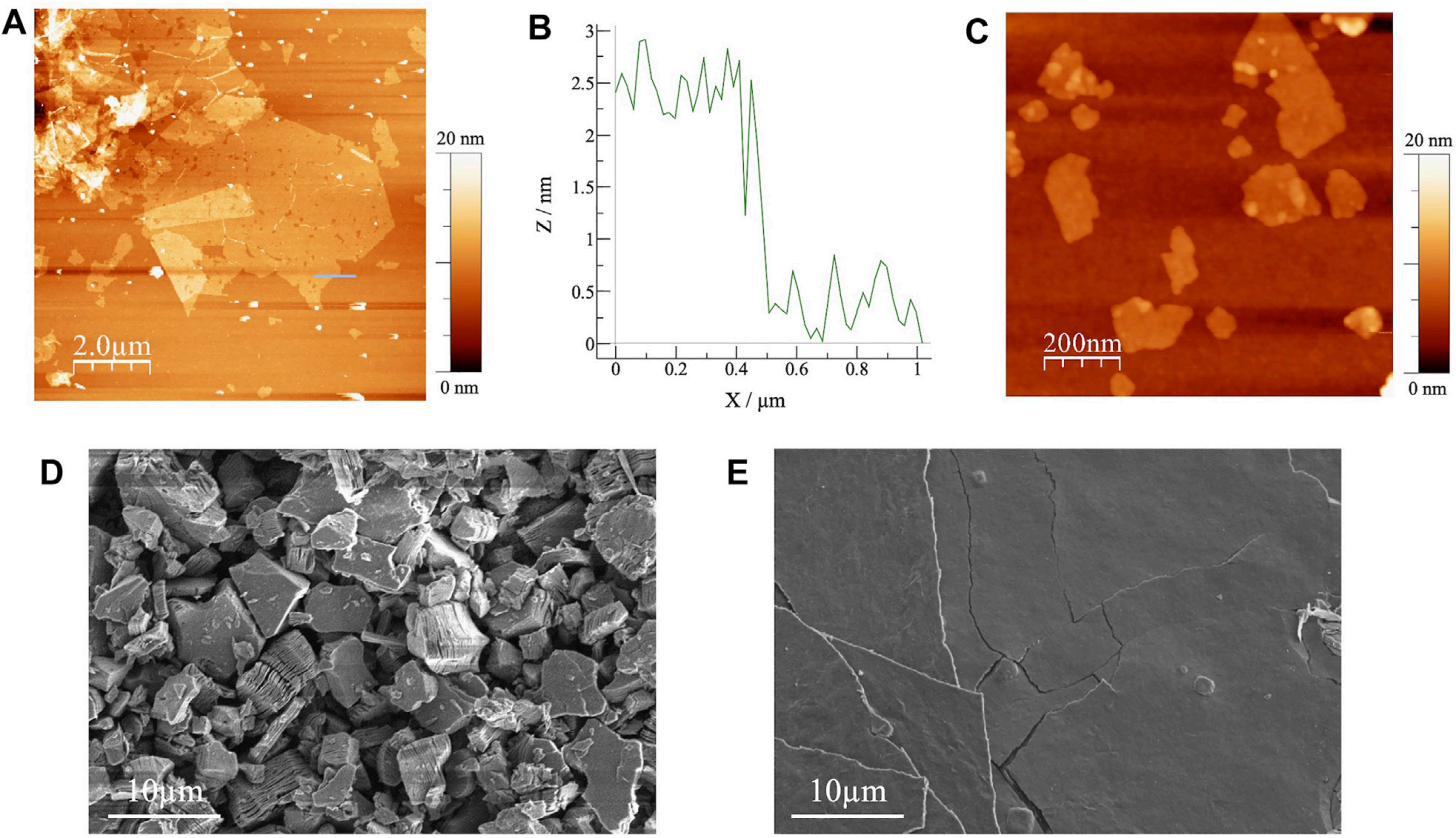
**(A)** AFM image and **(B)** the corresponding height profile of exfoliated Ti_3_C_2_T_*x*_ MXene without sonication; **(C)** AFM image of exfoliated Ti_3_C_2_T_*x*_ MXene prepared by the sonication treatment; SEM images of **(D)** pristine and **(E)** exfoliated Ti_3_C_2_T_*x*_ MXene.

Next, the exfoliated Ti_3_C_2_T_*x*_ MXenes prepared by the spin-coating method were characterized by AFM. A typical AFM image is shown in Figure 2A and the height profile is shown in Figure 2B. The MXene flakes were approximately 2 nm thick, consistent with previous measurements of single-layer MXene (Xuan et al., 2016). Without the sonication treatment, the exfoliated sample presented MXene sheets with an approximate size of >5 µm (Figure 2A), but after sonication, small flakes (<500 nm in size) were observed (Figure 2C). Because tiny defective pinholes were detected on the surface of the large MXene sheet (Figure 2A), it appeared that sonication proceeded by tearing the sheets into small pieces. In the macroscale film, the sheet resistance would be increased by the increased number of contact-resistance points between the networked MXene flakes.

Figure 2D shows a SEM image of pristine Ti_3_C_2_T_*x*_ MXene filtrated on the MCE membrane filter. Although the inter layer distances were slightly expanded, most of the flakes maintained the multilayer structure after the sonication treatment. Judging from the polycrystalline-like morphology, a conductivity network is not easily formed in this film. The lateral size of the multilayer Ti_3_C_2_T_*x*_ was 5–10 μm, compatible to that of exfoliated Ti_3_C_2_T_*x*_ in the AFM measurement. Conversely, an SEM image of the exfoliated Ti_3_C_2_T_*x*_ confirmed the formation of paper-like film structures (Figure 2E). In addition, larger domain films, compared with the observed lateral size in AFM, were obtained by the random stacking of the exfoliated flakes, which were similar to other two-dimensional nanomaterials (Dikin et al., 2007; Yun et al., 2017).

**FIGURE 3:**
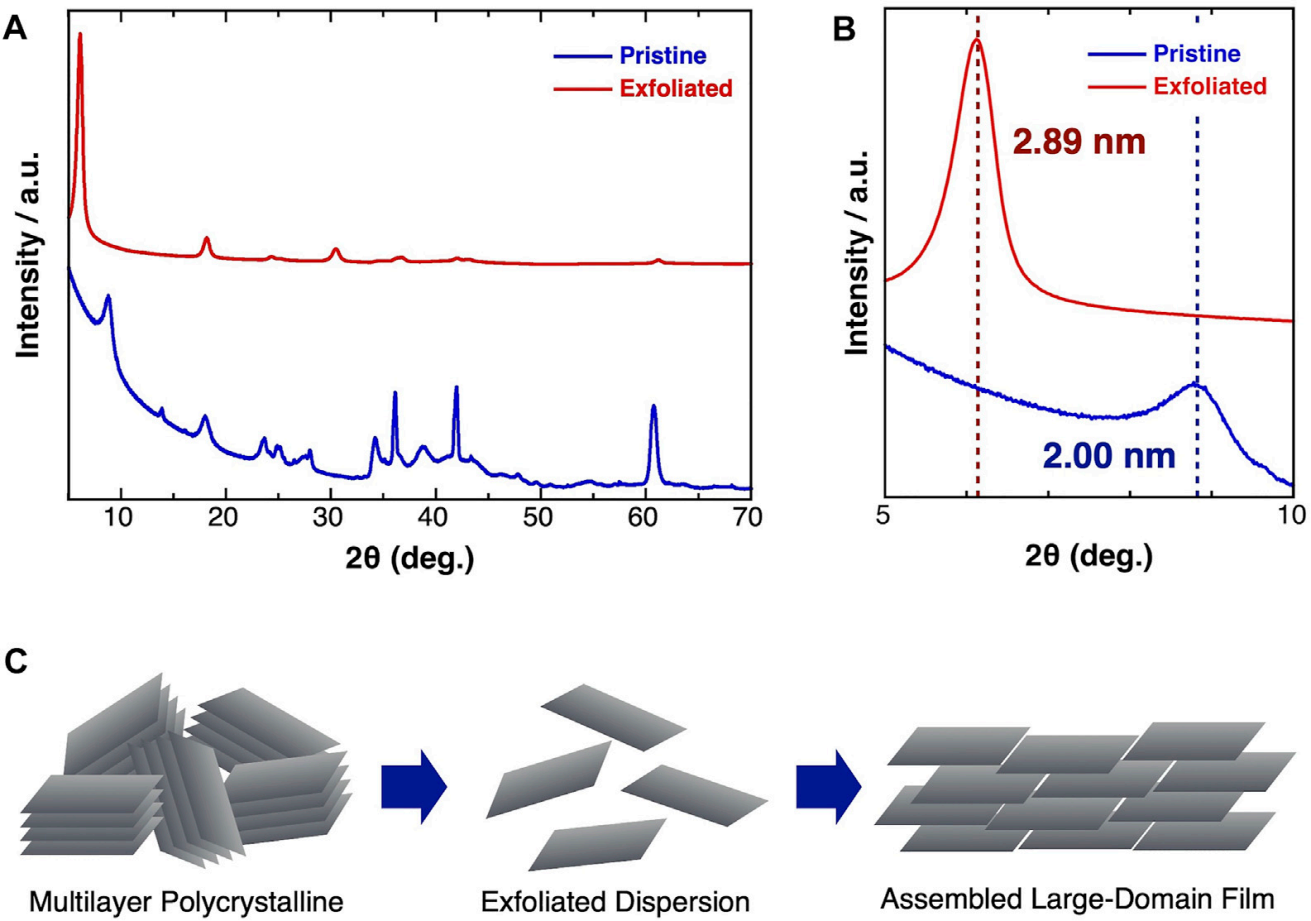
**(A)** Whole region and **(B)** magnified XRD pattern of (blue) pristine and (red) exfoliated Ti_3_C_2_T_*x*_ MXene. **(C)** Schematic image of the exfoliation process and the film formation process of the exfoliated MXene.

The detailed structural changes during the exfoliation and film formation processes were uncovered using XRD measurements (Figure 3). The XRD pattern of the pristine Ti_3_C_2_T_*x*_ was in good agreement with that of the HF-treated Ti_3_C_2_T_*x*_ (Naguib et al., 2011) (Figure 3A). After the exfoliation process, the (002) peak (2θ = 8.84°) shifted to a lower angle (2θ = 6.12°), corresponding to an expansion of the average inter layer spacing from 2.00 to 2.88 nm (Figure 3B). A similar expansion of the inter layer distance was reported in previous exfoliation studies using organic intercalation agents (Mashtalir et al., 2013; Naguib et al., 2015). Moreover, the relative intensity of the (002) peak in the spectrum of the exfoliated Ti_3_C_2_T_*x*_ was obviously enhanced, indicating that the single-layer flakes were stacked with a well-alignment. Figure 3C shows a schematic image of the exfoliation and film formation processes. The multilayer Al-residual Ti_3_C_2_T_*x*_ was exfoliated by the treatment with Me_4_NOH. Thereafter, the obtained single-layer Ti_3_C_2_T_*x*_ dispersion was reconstructed to assembled large-domain layered films, enabling excellent macroscale electric conductivity.

### Chemical Structure Characterizations

**FIGURE 4:**
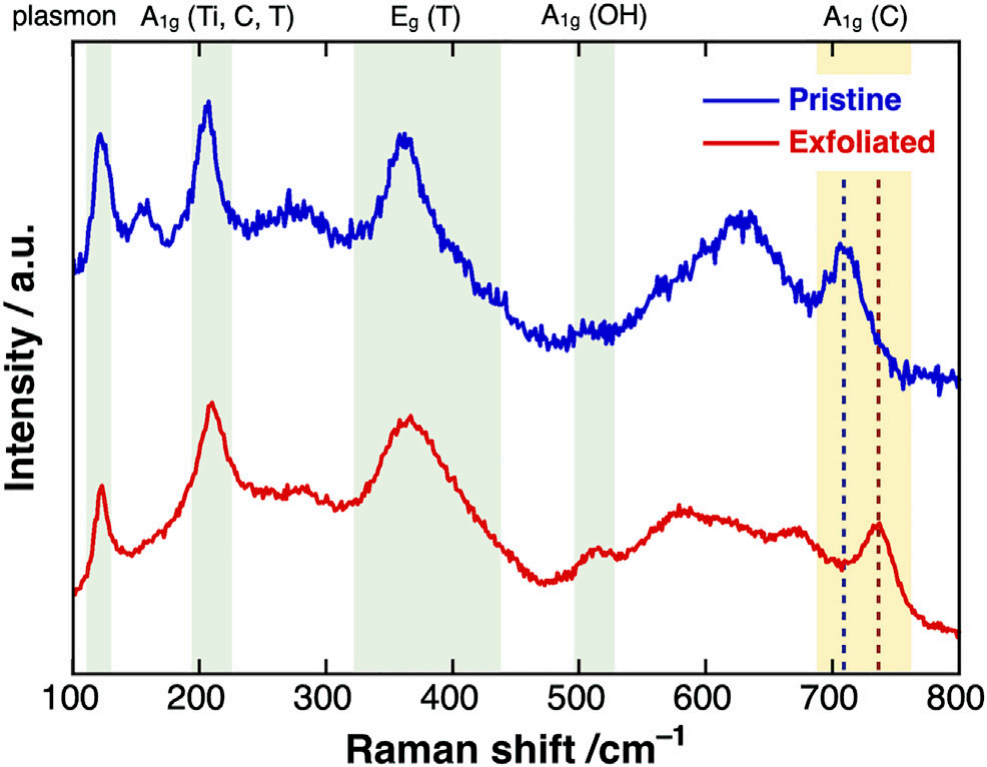
Raman spectra of (blue) pristine and (red) exfoliated Ti_3_C_2_T_*x*_ MXene.

We now report the Raman spectra of pristine and exfoliated MXenes excited by a 785-nm laser (Figure 4). The sharp peaks at 106, 204, and 380 cm^−1^ in both spectra were assigned to the plasmonic, A_1g_ (Ti, C, T), and E_g_ (T) peaks of Ti_3_C_2_T_*x*_ MXenes, respectively. After exfoliation, the A1g peak originating from carbon atoms shifted from 709 to 737 cm^−1^, close to the typical value of single-layer Ti_3_C_2_T_*x*_ MXene. This phenomenon strongly suggested that most of the exfoliated MXenes in the dispersion maintained the single-layer state (Sarycheva and Gogotsi, 2020). In addition, the intensity of the A_1g_ peak of the hydroxy groups at around 510 cm^−1^ and the full width at half maximum of the E_g_ (T) peak were slightly increased, implying that surface oxidation occurred during the etching reaction.

**FIGURE 5:**
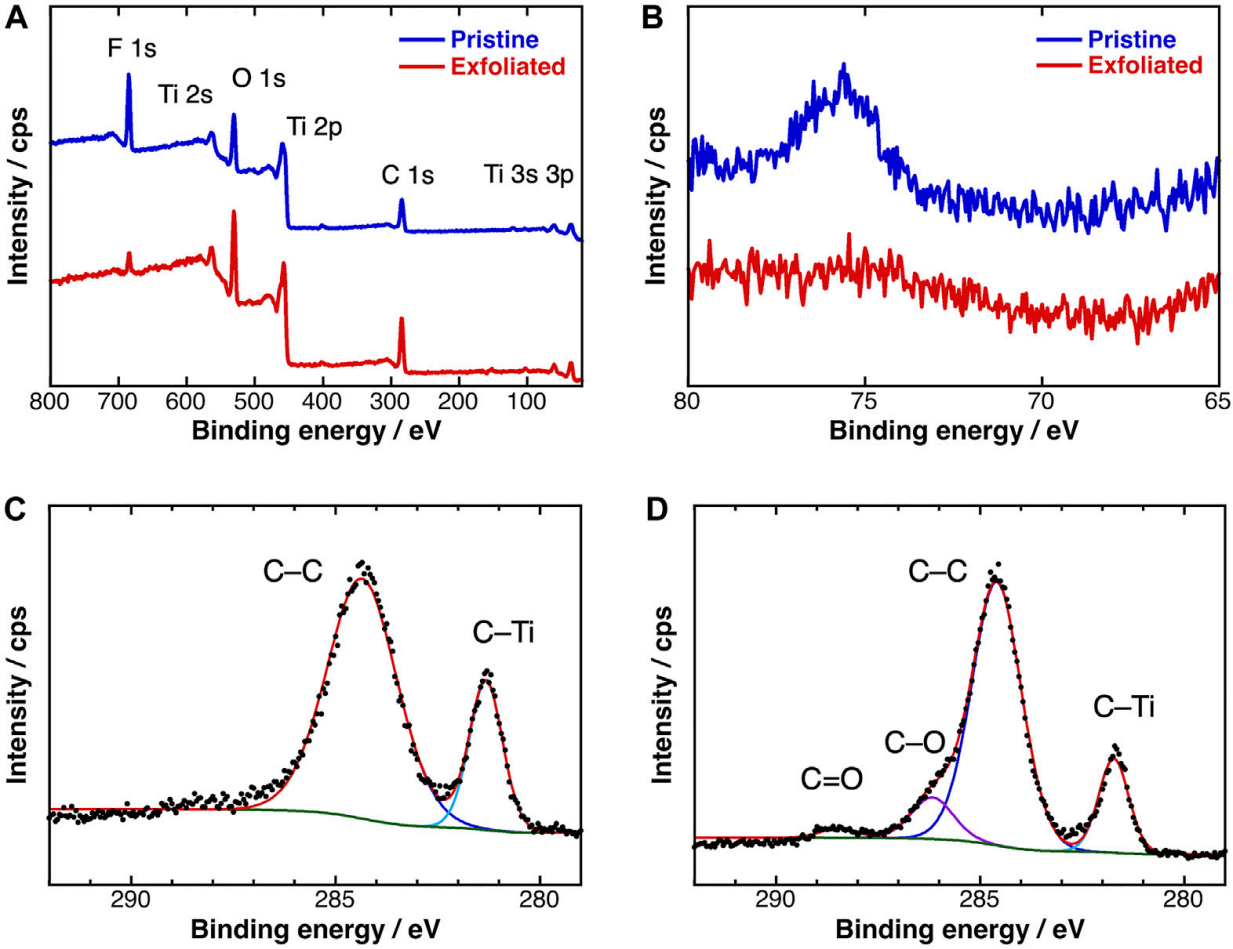
**(A)** Survey and **(B)** Al 2p XPS spectra of (blue) pristine and (red) exfoliated Ti_3_C_2_T_*x*_ MXene; C 1p XPS spectra of **(C)** pristine and **(D)** exfoliated Ti_3_C_2_T_*x*_ MXene.

The chemical structures of the MXenes were further clarified in XPS measurements. The survey spectra of pristine and exfoliated MXenes presented the distinct peaks of titanium (3p: 36 eV, 3s: 61 eV, 2p: 459 eV, and 2s: 563 eV), carbon (1s: 284 eV), oxygen (1s: 531 eV), and fluorine (1s: 691 eV) atoms (Figure 5A). The peak assigned to Al 2p was clearly absent in the high-resolution spectrum of the exfoliated sample (Figure 5B). As expected, the content percentage of aluminum atoms dropped from 1.6 to <0.1 at.% (Table 2). The F 1s peak was also depressed in the survey spectrum of the exfoliated sample. The intensity of the F 1s peaks drastically reduced from 21.5 to 5.4, indicating that the substitution reaction of Ti–F to Ti–OH bonds was promoted by the Me_4_NOH reagent. Meanwhile, the hydroxy group population increased, consistent with the behavior of the A_1g_ (OH) peak in the Raman spectrum.

**TABLE 2:** XPS atomic percentages of pristine and exfoliated Ti_3_C_2_T_*x*_ MXene.

Sample	Atomic content [%]				
	F 1s	O 1s	Ti 2p	C 1s	Al 2s
Pristine	21.5	22.9	18.8	35.2	1.6
Exfoliated	5.4	31.0	17.7	45.9	<0.1

Figures 5C,D display the HR C 1s spectra of the pristine and exfoliated MXenes, respectively. After separating the peaks, the major peaks at 281 and 284 eV were assigned to C–Ti and C–C bonds, respectively. In the spectrum of the exfoliated sample, the shoulder band at 286.2 eV originated from the carbon–oxygen single bond (C–O) of hydroxy groups, and the negligibly weak peak at 288.5 eV was attributable to carbon–oxygen double bonds (C=O). The generation of these oxidized carbon peaks suggest that partial chemical etching of the titanium atoms with Me_4_NOH exposed the inner carbon atoms. Such structural defects are consistent with the pinholes observed in the AFM image of the large-sized MXene sheets.

### Application to Flexible Transparent Conductive Films

**FIGURE 6:**
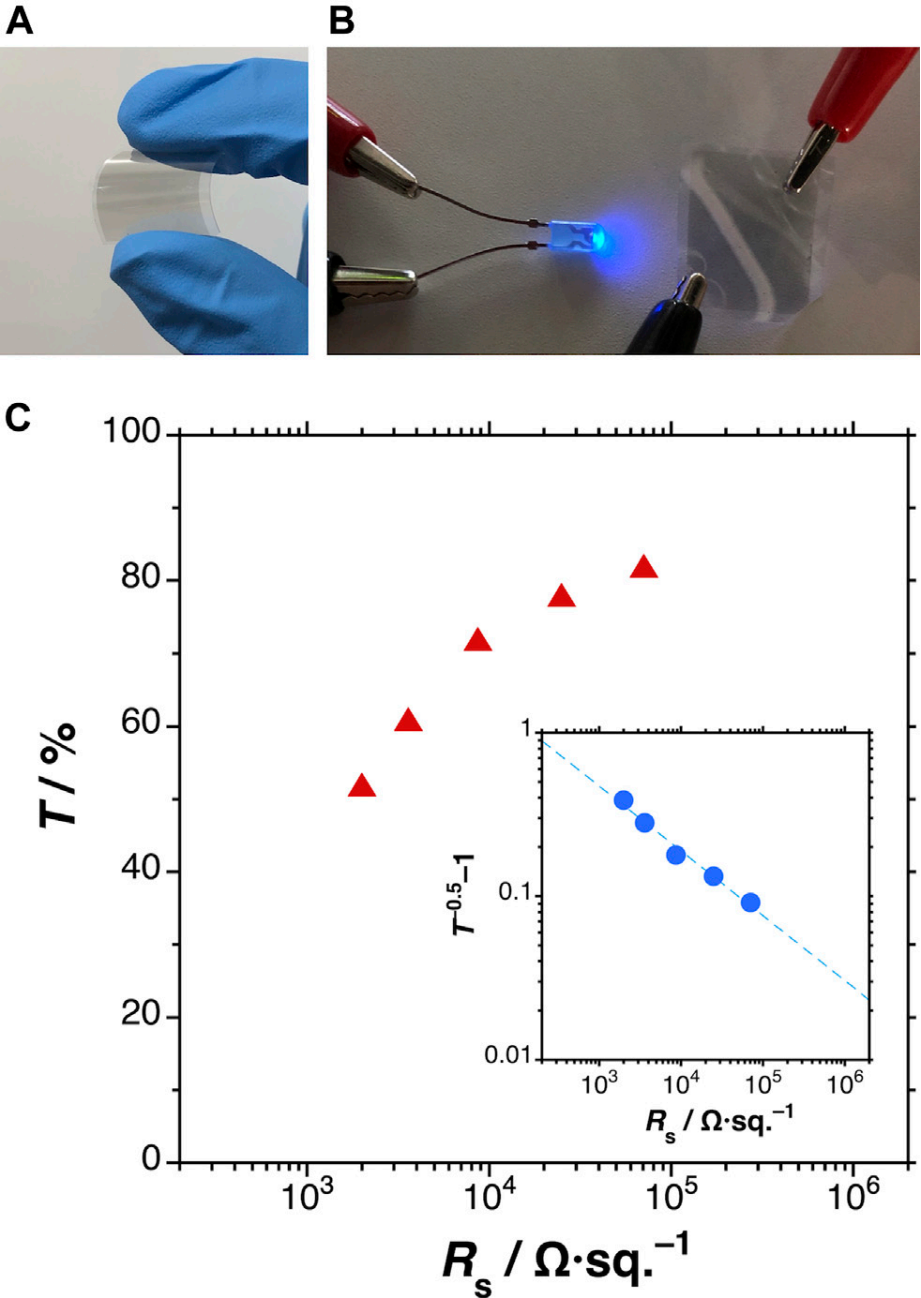
**(A**,**B)** Typical photographs of the fabricated MXene transparent conductive films; **(C)** plots of transmittance at 600 nm (main) and *T* ^−0.5^–1 (inset) versus average sheet resistance of the MXene films.

Finally, the transparent conductive MXene films were demonstrated in an application (Figure 6). The MXene films were formed on a PEN film by spraying of the dispersion diluted in methanol solvent. Figures 6A,B are typical photographs of the fabricated MXene films. The film was bendable (Figure 5A) and sufficiently electrically conductive (Figure 6B). Figure 6C plots the transmittance at a wavelength of 600 nm versus the average sheet resistance of various MXene films. The *R*_s_ at 78% transmittance was 2.5 × 10^4^ Ω/sq., comparable to those of previous spray-coated examples (Hantanasirisakul et al., 2016; Zhou et al., 2021). The inset of Figure 6C plots *T* ^−0.5^–1 as a function of *R*_s_. The resulting bulk-type conductivity curve is consistent with those of other MXene films (Dillon et al., 2016).

## Conclusion

In conclusion, we exfoliated Al-residual multilayered Ti_3_C_2_T_*x*_ MXene with Me_4_NOH in THF/H_2_O co-solvent. Ti_3_C_2_T_*x*_, exfoliated into single layers without sonication, as confirmed in AFM observations and Raman spectroscopy. The conductivity was 100+ times higher than in films exfoliated under previously reported aqueous conditions. Moreover, transparent conductive films were formed on flexible PEN substrates using the synthesized Ti_3_C_2_T_*x*_ dispersion. XPS measurements of the exfoliated samples clarified that the Al atoms were removed and the Ti–F bonds transformed into Ti–OH bonds. The generated hydroxy groups, which can convert to a variety of functional groups, expand the feasibility of MXene as a conductive support material in catalysis, battery, and electrochemical applications. Thiol (Omachi et al., 2020) or amino groups (Matsumoto et al., 2020) can immobilize the metal clusters and nanomaterials, realizing novel conductive nanohybrid materials rather than the existing graphite or graphene examples. Further chemical modification of Ti_3_C_2_T_*x*_ toward electrode applications in battery systems is ongoing in our laboratory.

## Data Availability

The original contributions presented in the study are included in the article/Supplementary Material, further inquiries can be directed to the corresponding authors.
